# Associations between Pathological Gambling and Psychiatric Comorbidity among Help-Seeking Populations in Hong Kong

**DOI:** 10.1100/2012/571434

**Published:** 2012-06-18

**Authors:** Daniel T. L. Shek, Elda M. L. Chan, Ryan H. Y. Wong

**Affiliations:** ^1^Department of Applied Social Sciences, The Hong Kong Polytechnic University, Hunghom, Hong Kong; ^2^Public Policy Research Institute, The Hong Kong Polytechnic University, Hong Kong; ^3^Department of Social Work, East China Normal University, Shanghai 200062, China; ^4^Kiang Wu Nursing College of Macau, Macau; ^5^Division of Adolescent Medicine, Department of Pediatrics, Kentucky Children's Hospital, University of Kentucky College of Medicine, Lexington, KY 40506, USA; ^6^Tung Wah Group of Hospitals Even Centre, Hong Kong

## Abstract

Problem gambling is complex and often comorbid with other mental health problems. Unfortunately, gambling studies on comorbid psychiatric disorders among Chinese communities are extremely limited. The objectives of this study were to (a) determine the prevalence of comorbid psychiatric disorders among treatment-seeking pathological gamblers; (b) compare the demographic profiles and clinical features of pathological gamblers with and without comorbid psychiatric disorders; (c) explore the associations between pathological gambling and psychiatric disorders and their temporal relationship. Participants (*N* = 201) who sought gambling counseling were examined by making Axis-I diagnoses including mood disorders, schizophrenia spectrum disorders, substance use disorders, anxiety disorders, and adjustment disorder. Results showed that 63.7% of participants had lifetime comorbid psychiatric disorder. The most common comorbid psychiatric mental disorders were mood disorders, adjustment disorder, and substance use disorders. Pathological gamblers with psychiatric comorbidities were significantly more severe in psychopathology, psychosocial functioning impairment, and gambling problems than those without the disorders.

## 1. Introduction

Pathological gambling can have a wide range of adverse effects on individuals, families, and society. The negative consequences include financial and debt problems, marital conflict, criminal behavior, family violence and breakdown, as well as severe emotional and mental health problems [1–3]. In Hong Kong, a study conducted in 2008 showed that the prevalence of probable problem gambling and pathological gambling was 2.2% and 1.8%, respectively [4]. These figures were comparable with other cities such as Macau (2.5% probable problem gambling and 1.8% pathological gambling [5]) and Singapore (2% probable problem gambling and 2.1% pathological gambling [6]).

In Hong Kong, the development of pathological gambling treatment and research is still at an early stage. One of the neglected areas in the literature is the comorbid conditions of pathological gambling. International studies have reported that pathological gambling was highly comorbid with mental health disorders such as anxiety disorders, depression and affective disorders, and substance use disorders [7–10]. There are research findings showing that patients with multiple diagnoses were more impaired and less responsive to treatment than those with a single diagnosis [11]. A number of epidemiological studies have been carried out to investigate the prevalence of comorbid mood disorders, anxiety disorders, substance use disorders, and other psychiatric disorders among pathological gamblers. Although comparisons between studies are difficult due to differences in samples, inclusion criteria, and assessment tools used, these studies generally showed a high psychiatric comorbidity among pathological gamblers identified from the general population sample [9, 12, 13].

Kessler and colleagues [12] reported a significant prevalence rate of 55.6% of mood disorders among pathological gamblers whereas Petry et al. [9] reported a prevalence rate of 49.6%. Regarding anxiety disorders, Petry and colleagues [9] found a prevalence rate of 41.3% among problem gamblers whereas Kessler et al. [12] reported an even higher rate of 60.3%. Besides, the risk of experiencing moderate/high severity gambling was shown to be 1.7 times higher for people with anxiety disorder. Regarding substance use disorders, Petry and colleagues [9] found alcohol use disorder among almost three quarters (73.2%) of pathological gamblers, whereas drug use disorder and nicotine dependence were found among 38.1% and 60.4% of pathological gamblers, respectively. El-Guevaly et al. [13] also reported that people with substance or alcohol dependence had 2.9 times higher risk having gambling problems compared to people without the disorder. Unfortunately, there is no data on comorbid schizophrenia spectrum disorders from existing epidemiological studies on pathological gamblers.

 Several studies have also reported a high prevalence of comorbid psychiatric disorders among pathological gamblers seeking treatment [8, 14, 15]. Ibáñez et al. [8] and Kausch [15] reported that 50% of treatment seeking pathological gamblers had a clinical diagnosis of mood disorders in their lifetime. Anxiety disorders are also common, with the lifetime prevalence estimated to range between 4.3% and 8.5% [8, 14, 15]. Kausch [15] reported a prevalence rate of 66.4% of substance abuse or dependence among treatment-seeking gamblers. The reported rate of comorbid alcohol abuse or dependence was 8.5% and 23.2% across studies [14, 15]. As for schizophrenia spectrum disorders, the prevalence rate reported was 4.3% to 6% [14, 15]. Based on these findings, it can be conjectured that psychiatric comorbidity has a high rate among pathological gamblers identified both in the community sample and treatment seeking sample.

Some researchers argued that the high comorbidity of psychiatric disorders among pathological gamblers suggested an association between these conditions [8, 9, 12, 13]. However, studies on their temporal relationships are limited. Researchers have generated a number of hypotheses regarding the pattern of temporal priority in onset. For example, some psychiatric disorders might be viewed as risk factors for pathological gambling, while others as consequences of pathological gambling [12]. Some researchers suggested that patients with major depressive disorder resorted to gambling as a means of escaping from the depressive symptoms. People with depressive symptoms often suffered from an underlying anhedonic state such as an inability to experience pleasure from normal pleasurable life events. Excessive gambling activity might offer an escape or a feeling of reward pursued to compensate the feeling of flat and lacking pleasure [16]. In contrast, other studies reported that depression observed in pathological gamblers was not primary to underlying gambling symptoms, but constituted a secondary reaction to the negative consequence of pathological gambling such as family breakdown or financial problems [17, 18].

Blum and colleagues [19] argued that there are underlying psychological mechanisms linking pathological gambling and other addictive behaviors. They proposed a concept of reward deficiency syndrome that links all the addictive, compulsive and impulsive behaviors. Under this hypothesis, persons at risk for one addiction could be viewed as having a higher risk for other addictions. Therefore, people with pathological gambling behavior are more likely to develop other addictive, compulsiveor impulsive, and dysfunctional behaviors. McCormick [20] suggested that a high degree of impulsivity and a high level of negative affection make pathological gamblers particularly vulnerable to feelings of helplessness and hopelessness. This idea was supported by the high rate of suicidal attempts among treatment-seeking pathological gamblers [15].

A search of the literature showed that very few studies had addressed the temporal relationship between pathological gambling and its cooccurring psychiatric disorders. It has been reported that the onset and maintenance of pathological gambling could be predicted by some preexisting psychiatric disorders such as anxiety, mood, and substance use disorders. At the same time, pathological gambling could predict the onset of generalized anxiety disorder, posttraumatic stress disorder, and substance dependence [12]. In another study on the temporal relationship between gambling and mood disorders [21], it was reported that 70% of participants had mood disorder before the development of their gambling problems. The onset of gambling behavior was found to be earlier in men with major depressive disorders than in women [21]. Individuals in the “mood first” group had higher rates of substance dependence while individuals in the group “gambling first” had higher rates of anxiety disorders like panic disorder and generalized anxiety disorder [21]. Studies on substance use reported that alcohol problems, tobacco use, and marijuana use tended to occur before gambling problems [22].

Kessler and colleagues [12] reported findings on the temporal relationship between psychiatric disorders and problematic gambling behaviors in their epidemiological study. Among people with lifetime comorbidity of pathological gambling and other psychiatric disorders, the onsets of mood disorder (65.1%), anxiety disorder (82.1%), and substance use disorder (57.4%) were reported to be earlier than the onset of pathological gambling [12]. Although published researches provided some data on the prevalence of psychiatric comorbidity and pathological gambling, little information was available regarding the cultural effects, temporal relationship, clinical as well as social significance of the observed conditions. Most of the available comorbidity research studies on pathological gambling were conducted on Caucasian study samples. There is a severe lack of studies on the prevalence and relationship of pathological gambling and mental health disorders and other comorbid conditions in the Chinese community. The lack of Chinese dominance sample studies may limit the applicability of the available findings.

Against the above background, this is the first study to report prevalence rates of comorbid psychiatric disorders among Chinese pathological gamblers in Hong Kong. This study will provide valuable data on the temporal relationships between psychiatric disorders and pathological gambling, severity of gambling problems and psychiatric symptoms, and level of functional impairment among Chinese treatment-seeking pathological gamblers in Hong Kong. The objectives of the current study were to: (a) determine the prevalence of comorbid psychiatric disorders among pathological gamblers seeking treatment in Hong Kong; (b) compare the demographic profiles and clinical features of pathological gamblers with and without comorbid psychiatric disorders; (c) assess the associations of pathological gambling with comorbid psychiatric disorders, as well as its temporal relationship.

## 2. Methods

### 2.1. Participants and Procedures

As a first step, an extensive literature review on the prevalence and relationship between psychiatric disorders and pathological gambling as well as their temporal relationship was conducted. Based on the research findings and clinical experiences, a comprehensive design of our study was formulated. All research personnel collecting the data in this study received training and supervision at Hong Kong Mood Disorders Centre on how to use the psychiatric research instrument (i.e., Chinese version of Structured Clinical Interview for DSM-IV: SCID) before conducting research interview.

The data collection period for this study was from June 2009 to February 2010. Inclusion criteria for the participants of this study were age 18 or older, and meeting five or more DSM-IV diagnostic criteria for pathological gambling. Exclusion criteria adopted in the study were manifestation of signs of cognitive impairments or imminent suicidal risk, as well as inability to read Chinese characters or to speak Cantonese. Clients who met the inclusion criteria and sought services from the Tung Wah Group of Hospitals Even Centre and Zion Social Services Yuk Lai Hin Counselling Centre within the data collection period were invited to join the study during the intake process. The purpose and procedure of this research and interview were explained to all eligible participants. Participants were reassured that all individual identifiable information would be treated as confidential and would not be released to anyone outside this research project. Written informed consent was obtained from participants prior to joining the study.

The participants were first provided with a self-administered questionnaire which attempted to collect their demographic data, gambling characteristics and psychiatric symptoms. They were then interviewed face-to-face by trained health professionals such as social workers or clinical psychologists who administered the SCID and other instruments to assess participants' psychiatric comorbidities, gambling problems, and functional impairment. Both parts of the self-administrated questionnaire and semistructured interview took about 1 hour and 30 minutes. Upon the completion of the interview, participants received a HK$100 supermarket voucher as an incentive. During the data collection period, a total of 201 interviews (182 men and 19 women) were successfully completed.

### 2.2. Instruments

The information collected from the participants is as follows.

#### 2.2.1. Demographic Information

The participants were asked to provide information about their current age, gender, marital status, educational attainment, living arrangement, type of housing, economic status, occupation, and personal income.

#### 2.2.2. Psychiatric Symptoms

The Brief Symptom Inventory (BSI) is a self-administered 53-item inventory used to measure respondents' psychiatric symptoms experienced in the previous week [23, 24]. Each item in the BSI is rated on a 5-point Likert scale ranging from 0 to 4, reflecting the intensity of distress experienced in the past week from “not at all present” to “extremely present.” These 53 items measure a variety of problems and complaints in nine primary symptom dimensions including depression, somatization, obsessive-compulsive, anxiety, psychoticism, hostility, phobic anxiety, interpersonal sensitivity, and paranoid ideation. Nine subscale scores are derived and profiled according to these dimensions.

In addition to the individual subscales, three global measures of psychological distress are derived from the BSI: General Severity Index (GSI), Positive Symptom Total Score (PST), and Positive Symptom Distress Index (PSDI). The GSI indicates the overall level of psychological distress, the PST reveals the number of symptoms endorsed, and the PSDI quantifies the average intensity of distress the participant experienced. Higher scores stand for greater severity of symptoms. Adequate reliability and validity has been demonstrated in the BSI [23, 25] and its Chinese version has shown good internal consistency in previous studies in Hong Kong [26, 27] and convergent validity for Chinese samples [28].

#### 2.2.3. Psychiatric Comorbidities

The Structured Clinical Interview for DSM-IV Axis I Disorders (SCID-I; [29]) is a semistructured instrument assessing major Axis I diagnoses described in DSM-IV [30]. The modules of the SCID-I selected for this research included mood disorders (modules A and D), schizophrenia spectrum disorders (modules B and C), substance use disorders (module E), anxiety disorders (module F), adjustment disorder (module I), and optional module (module J). The modules on somatoform disorders (module G) and eating disorders (module H) were not administered due to their lack of relevance to our study. Supplementary questions on nicotine dependence were added in the module of substance use disorders. Age at onset for any particular psychiatric disorder diagnosed was recorded to examine the temporal relationship between psychiatric disorders and pathological gambling. The current Chinese version of the SCID was developed in the 1970s and revised by The Chinese University of Hong Kong in 1996. The Chinese version of the SCID was found to be a reliable diagnostic instrument for the diagnosis of mood disorders, adjustment disorders, anxiety disorders, and schizophrenia spectrum disorders for outpatients [31, 32]. The non-patient version of SCID-I used in this study is largely similar to the patient version of SCID-I.

#### 2.2.4. Gambling Characteristics

The Addiction Severity Index (ASI)—Gambling Section—is a standardized and semistructured instrument which is widely used to evaluate the intake status for clients seeking substance use treatment [33, 34]. The instrument measures the severity of multiple domains which are commonly affected by substance misuse, such as medical, psychiatric, employment, family/social, legal, alcohol and drug problems in the previous month. The ASI has demonstrated excellent reliability and validity in various substance-abusing populations [33, 35], the severely mentally ill [36], as well as pathological gamblers seeking treatment [37].

In this study, the gambling section was developed as a supplement to the traditional ASI to assess the severity of gambling problems [38, 39]. Items related to the frequency and amount spent on gambling, number of days gambling more than was affordable, and the number of days experiencing gambling problems, perceived trouble towards the gambling problems, and perceived importance towards gambling treatment were calculated to derive composite scores with a value ranging from 0 to 1. Higher composite scores indicate a greater severity of gambling problems. The gambling subscale has also shown reliability and validity in populations of pathological gamblers [38–40].

#### 2.2.5. Severity of Nicotine Problems

The severity of nicotine dependence was assessed by a widely used measure, Fagerstrom Test for Nicotine Dependence (FTND) [41]. FTND has a revised scoring for Fagerstrom Tolerance Questionnaire (FTQ) which was developed to measure the dependency on nicotine [42]. The FTND includes six items, which are scored dichotomously and rankly, and produces a total score ranged from 0 to 10, with higher scores indicating greater severity of nicotine addiction. The FTND was translated into Chinese by the Bureau of Health Promotion of the Department of Health in Taiwan. Previous studies have demonstrated that the FTND, and its Chinese version, has shown satisfactory validity and reliability [41, 43].

#### 2.2.6. Level of Psychosocial Functional Impairment

The Range of Impaired Functioning Tool (LIFE-RIFT) is a valid and reliable semistructured instrument for assessing functional impairment [44]. It was derived from items that were included in the Longitudinal Interval Follow-up Evaluation (LIFE; [45, 46]). The LIFE-RIFT items focus on the assessment of functioning in various domains including work (with different scales for employment, household duties, and student work), interpersonal relations, global satisfaction, and recreation. The LIFE-RIFT requires some clinical judgment from a trained interviewer such that supplementary questions with the guidance of behavioral anchors would be asked before giving the ratings. Scores on each domain range from 1 to 5 with higher scores indicating poorer functioning. The total score of LIFE-RIFT was obtained by summing the subscale scores from four domains. Response of 0 (not applicable) and 6 (no information) were coded as missing items and would not be used for total score calculation. A total score would be derived only if there was no missing response in any of those four domains. In the “work” and “interpersonal” domains, the subscore would be the highest score among the multiple items addressing the domain (i.e., employment, household duties, and student work in “work” domain; interpersonal relations with spouse, children, relatives, and friends in “interpersonal relations” domain).

### 2.3. Data Analytic Strategies

Differences in demographic characteristics, gambling-related variables, and clinical correlates between pathological gamblers with and without comorbid psychiatric disorders were determined by independent sample *t*-tests for continuous variables (e.g., ASI), and chi-square tests for categorical variables (e.g., marital status). Chi-square was also used to examine prevalence estimates of pathological gambling and psychiatric comorbidities. Multiple regression analyses were then performed to investigate whether comorbid psychiatric disorders were predictive of severity of gambling problems. Presence of any comorbid psychiatric disorder and demographic characteristics were entered as fixed factors, while ASI composite score was entered as dependent variable, because young adults [47], male gender [38], being divorced or separated [48], lower education attainment [49, 50], being unemployed or on a low income [50] are risk factors for development of pathological gambling [47]. SPSS for Windows, version 17.0, was used to conduct all statistical analysis. All tests were two-tailed with statistical significance set at *P* < 0.05.

## 3. Results

Based on the DSM-IV operational definition, people who endorsed 5 or more symptoms were regarded as “pathological gamblers.” A total of 201 participants who met the criteria of pathological gambling were included in the present study, with a mean clinically rated DSM-IV score of 7.3. Among them, 128 participants (63.7%) had comorbid psychiatric disorders in their lifetime, whereas 90 participants (44.8%) had at least one comorbid psychiatric disorder at the time of evaluation.

Table 1 showed that about nine-tenths of 201 participants were men. Most of them were in the stages of early and middle adulthood, and reached secondary school level of educational attainment. Around 60% of the participants were married or cohabitating. Compared with those without current comorbid psychiatric disorder, results of chi-square test showed that a greater proportion of participants with current comorbid psychiatric disorder were divorced, separated, or widowed. Regarding the living arrangement, most of them lived with family members and there were a greater proportion of participants with current comorbid psychiatric disorder living alone than those without current comorbid psychiatric disorder.

In terms of economic status and personal income, most of the participants were employed on either a full-time or part-time basis, and earned within the range of HK$5,001 and HK$15,000 per month. Generally speaking, greater proportions of participants with both lifetime and current diagnosis of comorbid psychiatric disorders were unemployed than those without the psychiatric disorders. Moreover, greater proportions of participants with lifetime comorbid psychiatric disorder had no income than those without psychiatric comorbidities, but the difference was not found to be statistically significant in current diagnosis.

Table 2 showed the results for gambling behavior on both the lifetime and current diagnoses between participants with and without comorbid psychiatric disorders. It was found that most participants started gambling involving money in their early twenties, with the average age of 19.9. More than 80% of participants with both lifetime and current comorbid psychiatric disorders reported that they had debts at the time of study, compared to about 75% of participants without comorbid psychiatric disorders. *t*-tests analysis showed that there were significantly more participants with current psychiatric comorbidities with debt (86.7%) than those without current psychiatric comorbidities (74.8%), while comparison for lifetime diagnosis did not reach statistically significant level. On the other hand, severity of gambling problems assessed by ASI composite scores (which ranged from 0 to 1) with higher scores reflecting more severe gambling problems showed a mean ASI gambling scale composite score for participants was 0.38. For both lifetime and current diagnosis, the mean scores for participants with comorbid psychiatric disorders were significantly higher than those without the disorders, and reflecting greater severity of gambling problems (Table 2).

Psychiatric symptoms experienced by the participants in the past week were examined by the BSI. Participants with comorbid psychiatric disorders in general scored significantly higher than those without the disorders in all nine psychiatric symptom dimensions including depression, somatization, obsessive-compulsive, anxiety, psychoticism, hostility, phobic anxiety, interpersonal sensitivity, and paranoid ideation. Moreover, there were significant differences in the overall level of psychological distress, number of symptoms endorsed and the average intensity of distress experienced between participants with and without lifetime comorbid psychiatric disorders in general (Table 2).

The LIFE-RIFT was developed specifically to assess functional impairment with a range from 4 to 20. A higher score indicates more severe psychosocial dysfunction. Since 11 participants reported “not applicable” or “no information” for a particular domain of LIFE-RIFT, no total score could be calculated for these participants and they were coded as missing. Of the remaining 190 participants, results showed that participants with comorbid psychiatric disorders in general had significantly higher LIFE-RIFT score than those without the disorders (Table 2). Regarding nicotine problems, 104 (51.7%) participants reported that they ever smoked in their lifetime. Nine of them reported they were former smokers whereas 95 reported to be current regular tobacco users. Participants with comorbid psychiatric disorders in general had significantly higher FTND score than those without comorbid psychiatric disorders (Table 2).

Table 3 summarizes the prevalence rates for lifetime and current comorbidity of DSM-IV Axis I psychiatric disorders, including mood disorders, schizophrenia spectrum disorders, substance use disorders, anxiety disorders, and adjustment disorders, whereas Table 4 shows the number of comorbid psychiatric disorders possessed by pathological gamblers. All disorders were screened by detailed and structured interview of SCID by trained social workers or clinical psychologists. In Table 3, lifetime comorbid psychiatric disorders included mood disorders (*n* = 59; 29.4%), schizophrenia spectrum disorders (*n* = 5; 2.5%), substance use disorders (*n* = 62; 30.8%), anxiety disorders (*n* = 19; 9.5%), and adjustment disorders (*n* = 42; 20.9%). The most common comorbid psychiatric disorders for lifetime diagnosis were nicotine dependency (*n* = 49; 24.4%), major depressive disorder (*n* = 43; 21.4%), adjustment disorders (*n* = 42; 20.9%), and alcohol abuse or dependency (*n* = 23; 11.4%).

Similarly, current comorbid psychiatric diagnoses included mood disorders (*n* = 43; 21.4%), schizophrenia spectrum disorders (*n* = 3; 1.5%), substance use disorders (*n* = 46; 22.9%), anxiety disorders (*n* = 17; 8.5%), and adjustment disorders (*n* = 28; 13.9%). Similar to the results for lifetime diagnosis, the most common current psychiatric comorbidities were major depressive disorders (*n* = 30; 14.9%), nicotine dependency (*n* = 41; 20.4%), adjustment disorders (*n* = 28; 13.9%), and alcohol abuse or dependency (*n* = 16; 8.0%).

In Table 3, for those 128 participants with lifetime comorbid psychiatric disorders, 55.8% of them reported that the onset of psychiatric disorder was prior to the onset of pathological gambling. With respect to specific disorders, most of them reported the onset of any substance use disorders (74.6%) and anxiety disorders (57.9%) prior to the onset of pathological gambling, whereas the onset of mood disorders (62.7%) and adjustment disorders (64.3%) was later than the onset of pathological gambling.

As shown in Table 4, about two-thirds of participants (*N* = 128, 63.7%) had at least one lifetime Axis I disorder other than pathological gambling. Among them, 73 participants had one lifetime comorbid disorder, 33 participants had at least two disorders, and 22 participants had three disorders or more in their lifetime. For those participants who had lifetime comorbid psychiatric disorders, the average number of psychiatric comorbidity was 1.7. On the other hand, 90 participants (44.8%) had at least one current Axis I disorder other than pathological gambling. Among them, 69 participants had only one current comorbid psychiatric disorder, 12 of them had two disorders, and 9 of them had three disorders or more. For those participants who had current comorbid psychiatric disorders, the average number of psychiatric comorbidity was 1.36 (Table 4).

Previous research findings showed that participants with comorbid psychiatric disorders had significantly greater severity in gambling problems and psychiatric symptoms, level of impairment in psychosocial functioning as well as nicotine problems. Further analysis of the relationship between these clinical correlates and the presence of comorbid psychiatric disorder was conducted. Multiple regression analyses were then performed to investigate whether comorbid psychiatric disorders were predictive of severity of gambling problems (Table 5). The ASI was treated as the outcome variable, and presence of any comorbid psychiatric disorder, gender, education, and income were regarded as predictors. For both lifetime and current diagnosis, results of multiple regression analyses showed that the association between psychiatric comorbidity and gambling severity (ASI composite score) remained significant after adjusting for other demographic variables (lifetime morbidity: beta = 0.239, *P* < 0.001; current morbidity: beta = 0.300, *P* < 0.001).

## 4. Discussion

This study provides valuable information for clinical practice as well as research directions in future. In particular, it provides original findings on comorbid psychiatric disorders among pathological gamblers in Hong Kong using a clinical sample and structured diagnostic instruments to assess the prevalence of comorbid psychiatric disorders.

The present study showed that psychiatric comorbidity was related to the socio-demographic characteristics of pathological gamblers. Regarding marital status, compared with those without current comorbid psychiatric disorders, those with current comorbid psychiatric disorders had a significantly lower proportion of married/cohabitating status, but a greater proportion being single, divorced, separated, or widowed. This result was consistent with the characteristic of living arrangement that participants with comorbid psychiatric disorders had a relatively higher proportion of living alone than those without comorbid psychiatric disorders. One observation of these findings was that participants with comorbid psychiatric disorders had comparatively weaker supportive networks from family or the society and could experience emotional loneliness and a sense of helplessness. This result is consistent with the Western literatures that prior psychiatric disorders were associated with a substantially higher risk of divorce [51], and higher comorbidity rates of mood and anxiety disorders were found in individuals who were living alone [52].

With respect to the economic status and personal income of pathological gamblers, those with lifetime psychiatric comorbidities had significantly greater proportions of being unemployed and having no income than those without psychiatric comorbidities. The effects of a psychiatric disorder may cause significant distress or impairment in social, occupational, or other important areas of functioning. This further leads to an inability to fulfill work tasks and consequential financial hardship. The results supported the previous study which suggested that work impairment was one of the adverse consequences of psychiatric disorders [53].

In our study group, pathological gamblers with psychiatric comorbidities displayed more severe gambling problems than those without psychiatric disorders in general. They received a higher composite score in ASI gambling subscale and a much greater proportion of percentage for those with comorbid psychiatric disorders in general had a debt problem. These findings are consistent with previous studies on pathological gamblers in Western cultures showing that the existence of comorbid psychiatric problems was associated with more severe gambling behavior and longer term of gambling problems [8, 16, 54, 55]. One possible explanation for the findings is that the effects of comorbid psychiatric disorders may contribute to the persistence of pathological gambling. For example, some depressive symptoms such as diminished interest in anything or indecisiveness might lead to the increased use of maladaptive coping strategies such as excessive gambling to escape from the underlying anhedonic state. This pattern will form a vicious cycle that could complicate and hinder the treatment and recovery process for clients with both disorders.

Consistent with the previous study conducted by Ibáñez and his colleagues [8], the presence of comorbid disorders was associated with greater gambling severity as assessed by ASI scale. That means that multiple and recurrent comorbid psychiatric disorders might increase the severity in gambling. The results support the associations between psychiatric disorders and pathological gambling in which they might be counterproductive to each other and might intensify the gambling problem and the comorbid condition. For example, some participants in our study reported that they tended to engage in excessive smoking when they were involved in gambling activities. The previous study also revealed that daily tobacco smoking in pathological gamblers was common and associated with more severe gambling and financial problems [56]. Some participants reported that they tended to spend a longer time and a greater amount of money on gambling activities in order to deal with depressed moods and to pursue excitement. This is consistent with previous results that excessive gambling activity may offer an escape or a feeling of reward for depressed individuals, in order to compensate for their feeling of flat and lacking pleasure [16]. The findings of the current study suggest that comorbid psychiatric disorders could intensify pathological gambling behavior and produced further negative impacts on the individuals.

In terms of the clinical correlates, pathological gamblers with lifetime and current comorbid psychiatric disorders reported more severity in psychopathology, impairment in psychosocial functioning and nicotine problems compared to those without lifetime and current psychiatric disorder, respectively. For example, they had higher scores on BSI's nine dimensions—depression, somatization, obsessive-compulsive, anxiety, psychoticism, hostility, phobic anxiety, interpersonal sensitivity, and paranoid ideation. They also reported higher overall level of psychological distress, more psychiatric symptoms endorsed, and more intense distress experienced. These results are in line with previous comorbidity studies that pathological gamblers with comorbid psychiatric disorders had higher severity of depression and more trait and state anxiety [8]. In addition, pathological gamblers with psychiatric comorbidity experienced more severe symptoms of paranoid ideation, obsessive-compulsive thoughts and behaviors, hostility, interpersonal sensitivity or psychoticism. This pattern suggested that they may be more withdrawn and isolated, may feel inferior, and experience more distorted thinking initiated by delusional or projective thoughts. This study also showed that participants with comorbid psychiatric disorders were markedly impaired in at least one major areas of functioning such as occupational or academic achievement and interpersonal relations. These findings are consistent with the previous studies that people with multiple disorders were more impaired than those with a single diagnosis [57]. With respect to the comparison of severity of nicotine problems between groups, pathological gamblers with comorbid psychiatric disorders in general had significantly higher scores on FTND than those without comorbid psychiatric disorders. That means pathological gamblers with comorbid psychiatric disorders had experienced more severe nicotine problems.

Consistent with previous prevalence studies on psychiatric comorbidity, the current study showed that there were high prevalence rates (63.7% for lifetime diagnosis and 44.8% for current diagnosis) and wide ranges of comorbid psychiatric disorders among pathological gamblers seeking treatment. It has been well demonstrated in both community and clinical sample studies that pathological gamblers have high prevalence rates of comorbid mood disorder, anxiety disorder, and substance use disorders [8, 9, 12–15]. Similar findings were found in our study with the most common psychiatric comorbidities reported including major depressive disorders, adjustment disorders, nicotine dependency, and alcohol abuse or dependency for both lifetime and current diagnosis.

Among the participants who suffered from lifetime comorbid mood disorder, most of them suffered from major depressive disorder (21.4%), dysthymic disorder (6.5%), and bipolar disorder (2.0%). This is consistent with the previous literature showing a close link between pathological gambling and mood disorders [7, 48]. Around 63% of pathological gamblers with comorbid mood disorders reported that their mood disorders appeared after pathological gambling. This observation could be explained by the fact that some pathological gamblers suffered from depressive symptoms, such as loss of interests and pleasures, as a consequence of financial and psychological distress associated with gambling.

However, about one-fourth of the participants with comorbid mood disorders in our study reported that the onset of mood disorders was prior to the onset of pathological gambling. The findings suggested that gambling in some cases could be underlying to mood disorders while the longitudinal clinical course of pathological gambling may be influenced by the clinical course of an underlying mood disorder [54]. It was not uncommon for an individual with depression to develop pathological gambling as a way of escaping from and coping with depressive symptoms. Excessive gambling would provide them with feelings of reward and excitement which they repeatedly pursued to compensate for their flattened mood [16].

There are very few comorbidity studies on the prevalence rates of schizophrenia spectrum disorders among pathological gamblers in both community and clinical samples. In line with the findings of previous studies [7, 14, 15], the current study found a very low rate of lifetime comorbid schizophrenia spectrum disorder (2.5%). Previous studies suggested that genetics, early environment, neurobiology, and psychological and social processes were important factors in schizophrenia spectrum disorders while the onset typically occurs between the late teens and the mid-1930s [30]. Due to the low prevalence rates reported in our study and the inconclusive findings in the literature, there is little evidence to suggest that pathological gamblers were more likely to develop any schizophrenia spectrum disorder, or vice versa. Petry [58] argued that results from treatment-seeking samples may underestimate the association of pathological gamblers with severe mental health problems such as schizophrenia, since they may be less likely to receive treatment for another coexisting disorder such as pathological gambling. Therefore, further studies to examine pathological gambling among patients diagnosed with schizophrenia spectrum disorders are needed.

Results from previous studies on clinical samples have indicated that substance use disorders were prevalent in treatment seeking pathological gamblers with lifetime prevalence rates ranging from a quarter to two-thirds across studies [8, 14, 15]. The current study similarly showed the high lifetime prevalence rates for substance use disorder (30.8%) in pathological gamblers. The high prevalence rates of nicotine (24.2%) and alcohol use disorder (11.4%) among pathological gamblers could be explained by the fact that as alcohol consumption and tobacco use were not prohibited in gambling venues like casinos, it is common for pathological gamblers to engage in excessive drinking and smoking as integral parts of gambling experience. They may also model such behavior from other gamblers. Blum and associates [19] suggested that there were similar underlying psychological mechanisms associated with different drives such as pathological gambling and substance use. Thus, individuals with an addiction would have a higher tendency to develop other addictive, compulsive, or impulsive dysfunctional behaviors.

Regarding the early onset of substance use disorder among the participants with comorbid psychiatric disorders, almost three-quarters of them stated that onset of substance use disorders preceded the development of pathological gambling. Blum and colleagues [19] hypothesized a shared underlying psychological mechanism between substance abuse and pathological gambling linking all the addictive, compulsive and impulsive behaviors. Thus, individuals with one addiction could be viewed as being at a higher risk for another kind of addiction. With reference to this hypothesis, people with substance use disorder are more likely to develop other addictive and dysfunctional behaviors such as pathological gambling. Further studies to examine how much a person's cognitive functioning may be affected under the influence of substance use during the process of gambling will be helpful in the development of responsible gambling policy.

Our results on the lifetime prevalence rate of anxiety disorders (9.5%) corroborate earlier clinical sample studies which have generally found that pathological gamblers seeking treatment have a prevalence of lifetime anxiety disorders of less than 10%. However, the prevalence rates of specific types of anxiety disorder in the current study were generally lower than those reported in previous studies. For example, the current study showed lifetime-generalized anxiety disorder (GAD) rates of 2.0%, which was much lower than the 7.2% reported by Ibáñez et al. [8], and 40% by Black and Moyer [7]. Black and Moyer [7] also reported a prevalence rate for panic disorder of 10% whereas our study only revealed a rate of 2.5%.

A relatively high prevalence rate of current adjustment disorder (13.9%) was found in our study, which was slightly lower than the current rate of 17.4% reported in the previous study conducted by Ibáñez and his associates [8]. According to the descriptions of DSM-IV [30], one of the important features of adjustment disorder is that there is an identifiable stressor that develops clinically significant emotional or behavioral symptoms in an individual, in which the stressor is significantly indicated by marked distress in excess of what would be expected or by significant impairment in social or occupational functioning. In our samples of pathological gamblers seeking treatment, most of them reported that their significant psychological distress was due to specific negative life events. These life events experienced by pathological gamblers were significantly related to gambling which exhibited deleterious effects on their lives. The adverse consequence of excessive gambling activities included marital and family problems and breakdown, job loss, unmanageable debt, and legal problems that easily led to depressed mood and anxiety. This explanation is supported by the temporal relationship between onset of adjustment disorder and pathological gambling in our study, with 60.7% of pathological gamblers with current comorbid adjustment disorders reporting that adjustment disorders appeared after pathological gambling.

## 5. Recommendation and Conclusion

The present findings showed that pathological gambling was significantly associated with the presence of comorbid psychiatric disorders among pathological gamblers seeking treatment. In Hong Kong, pathological gambling is treated in specialized gambling counseling centres mainly staffed by social workers, and counselors. Lesieur and Blume [38] reported that it would be more effective to treat individuals with dual mental health problems and addictions such as pathological gambling and alcohol abuse simultaneously to avoid encumbrance of treatment progress. To accomplish the above objective, it is necessary to achieve a joint effort from helping professionals in both gambling counseling services and mental health services and to develop accurate assessment measures. Based on the findings of the study, recommendations of the study are outlined in the following paragraphs.

### 5.1. Use of an Integrative Approach Involving a Multidisciplinary Team

To ensure both pathological gambling and comorbid psychiatric disorders are treated effectively, it is recommended to establish a multidisciplinary team which includes professionals such as psychiatrists, clinical psychologists, social workers, and gambling counselors. Multidisciplinary professionals can work closely and collaboratively to formulate treatment plans and to attend regular case conferences in order to monitor clients' progress and adjust the treatment plans and strategies when necessary. A multidimensional and holistic treatment approach should also be used which include psychotherapy and counseling, medication, group counseling, financial education, family therapy, psychoeducation, relapse prevention, vocational counseling, and continuing care. There is a need to strengthen multidisciplinary and cross-sectional collaboration in the treatment of addictions such as pathological gambling. Using substance abuse as an example, there are substance abuse clinics under the Hospital Authority where patients with both substance abuse and other psychiatric problems are treated. In contrast, there is no such service for pathological gamblers.

### 5.2. Professional Training on Psychiatric Issues for Gambling Counselors

Treatment-seeking pathological gamblers with comorbid psychiatric disorders reported increased severity of gambling problems, psychiatric symptoms and functional impairment than those without comorbid psychiatric disorders. As such, early detection can minimize the risk of further harmful effects, reduce prolonged health care costs, and optimize treatment outcomes. Hence, it is important for counselors to be sensitive to psychiatric symptoms during the intake and treatment process as most of the gamblers would focus on the presenting problems related to gambling. It is recommended to provide professional training on psychiatric issues to gambling counselors with knowledge for early detection.

### 5.3. Comprehensive Assessment during the Intake Process

In order to establish an appropriate treatment program for pathological gamblers with psychiatric comorbidities, it is recommended to adopt a comprehensive intake assessment during the admission process. Some semistructured or structured psychiatric diagnostic interviews such as Structural Clinical Interview for DSM-IV (SCID; [29]) and Composite International Diagnostic Interview (CIDI; [59, 60]) are some of the available diagnostic assessments on comorbid psychiatric disorders for pathological gamblers.

### 5.4. Differentiation of Primary or Secondary Disorder to Inform Treatment Priority

In the present study, the temporal relationship between pathological gambling and comorbid psychiatric disorders was examined. This contributes to our understanding on the etiological associations between pathological gambling and comorbid psychiatric disorders among pathological gamblers. Winters and Kushner [61] pointed out that there are several ways to conceptualize this etiological association since pathological gambling could serve as either the cause or consequence of comorbid disorders. They also highlighted some general clinical guidelines which have been derived from previous comorbidity studies. It was suggested that the sequence of treating addictions such as pathological gambling and comorbid psychiatric disorder could depend on the severity of active psychiatric problems. If psychiatric symptoms of a client are not assessed as severe, counselors could first treat their pathological gambling while continuously observing the psychiatric symptoms. Until the client is no longer experiencing the distress as a result of pathological gambling, reassessment of psychiatric comorbidity could be conducted to inform the need for separate treatment for the comorbid psychiatric disorder. Nunes and his associates [62] further recommended treating the comorbid psychiatric disorder while managing gambling behavior concomitantly through a series of psychoeducational and behavioral modifications on the problem gambling behavior. It is believed that conjoint treatment strategy will be beneficial to pathological gamblers who use gambling as a way of coping with their psychiatric symptoms.

### 5.5. Collaboration between Mental Health Services and Pathological Gambling Services

Joint efforts from mental health services and pathological gambling services are necessary to ensure that individuals with dual diagnosis of pathological gambling and psychiatric disorders would be treated effectively. It is recommended that gambling counseling centres could collaborate with other mental health agencies to implement a wide range of new and enhanced services including individual and group counseling, workshops, community programs, screening protocol, and referral system.

### 5.6. Community Education of the Relationship between Pathological Gambling and Psychiatric Disorders

There is a need to increase public awareness of the intimate link between psychiatric problems and pathological gambling. Such understanding may have two important effects. First, the moral labeling of pathological gamblers will be reduced. Second, relatives and friends of individuals who suffer from mental disorders can pay particular attention to the gambling behavior. They should note that gambling is not a good coping strategy for mental patients. Psychoeducation on the risk of using gambling to cope with emotional and mental health problems is vital.

### 5.7. Establishment of an Addiction Practice, Research, and Training Centre in the Asia Pacific Region

It is common for clinicians and researchers to treat different excessive behaviors, such as pathological gambling and substance abuse, as distinct disorders, but evidence from recent literatures and clinical experiences started to support the view that many commonalities occur across different expressions of addictions. Shaffer and colleagues [63] proposed the syndrome model of addiction that a distinctive addiction might express the same underlying addiction syndrome and reflect shared etiology. Thus, addiction should be understood as a syndrome with multiple opportunistic expressions. In order to facilitate evidence-based practice and to link research, practice, and training collectively in the Asia Pacific region, it is recommended to establish an Asia Pacific addiction centre. The centre can create a platform for coordinating addiction research, practice, and professional training in an integrated fashion that will help researchers and clinicians better understand and provide effective treatment. Moreover, the centre can develop international linkage with other addiction services and academic institutions in Hong Kong and in the Asia Pacific region to advance local knowledge and to encourage interdisciplinary collaborations in the field of addictions.

There are several limitations of the present study. First, the cross-sectional nature and retrospective design of the present study does not allow us to understand the causal relationship between pathological gambling and comorbid psychiatric disorders. Future studies using a longitudinal course of gamblers would help to further clarify the temporal priority and causal relationship between these disorders, and help understand how comorbid psychiatric disorders affect the gambling treatment outcomes. Second, as a nonrandom clinical sample was used, participants seeking gambling treatment may have higher level of emotional distress and functional impairment. Therefore, whether pathological gamblers in the community show different characteristic is a question remains to be examined. Third, as information on psychiatric morbidity was based on the self-report data of the participants, there is no access to medical records. In future, collaboration with relevant psychiatric services can give a fuller picture of the problem areas. Finally, due to limited time and financial resources, not all psychiatric disorders were assessed in the present study. Other disorders such as conduct disorder (CD), attention deficit/hyperactivity disorder (ADHD), and personality disorders to be associated with pathological gambling in previous studies [9, 12] will remain for further studies. Despite the above limitations, the present study is an important addition to the literature.

## Figures and Tables

**Table 1:** Differences between pathological gamblers with and without lifetime and current psychiatric disorders in demographic characteristics.

Variable	Lifetime psychiatric disorders					Current psychiatric disorders				
	With MI (*N* = 128)	Without MI (*N* = 73)	*χ* ^2^	df	*P*	With MI (*N* = 90)	Without MI (*N* = 111)	*χ* ^2^	df	*P*
Demographic characteristics										
Gender										
Male	89.8%	91.8%	0.204	1	0.652	86.7%	93.7%	2.867	1	0.090
Female	10.2%	8.2%				13.3%	6.3%			
Age										
20 or below	0.8%	1.4%	0.268	4	0.992	1.1%	0.9%	0.996	4	0.915
21–30	18.8%	17.8%				16.7%	19.8%			
31–40	27.3%	28.8%				30.0%	26.1%			
41–50	29.6%	30.1%				27.7%	31.5%			
51 or above	23.4%	21.9%				24.4%	21.6%			
Marital status										
Never married	28.9%	28.8%	4.266	2	0.179	27.8%	29.7%	9.069	2	0.011
Married	56.3%	65.8%				53.3%	64.9%			
Widowed, separated or divorced	11.4%	5.5%				18.9%	5.4%			
Education										
Lower secondary school or below	28.1%	17.8%	3.436	2	0.179	30.0%	19.8%	3.622	2	0.163
Upper secondary school	42.2%	42.5%				42.2%	42.3%			
Sixth form or above	29.7%	39.7%				27.8%	37.8%			
Economic status										
Employed	73.4%	90.4%	8.297	2	0.016	70.0%	87.4%	9.437	2	0.009
Homemakers, retired, or students	6.3%	2.7%				6.7%	3.6%			
Unemployed	20.3%	6.8%				23.3%	9.0%			
Personal income										
$0	17.2%	8.2%	14.160	7	0.048	18.9%	9.9%	11.487	7	0.119
$1–$5,000	8.6%	15.1%				7.8%	4.5%			
$5,001–$10,000	21.9%	21.9%				22.2%	21.6%			
$10,001–$15,000	24.2%	24.7%				21.1%	27.0%			
$15,001–$20,000	14.1%	13.7%				17.8%	10.8%			
$20,001–$25,000	3.9%	11.0%				3.3%	9.0%			
$25,001–$30,000	3.9%	4.1%				3.3%	4.5%			
$30,001 or above	6.3%	15.1%				5.6%	12.6%			
Living arrangement										
Living alone	12.5%	4.1%	5.195	3	0.158	15.5%	4.5%	9.467	3	0.024
With spouse	14.1%	20.5%				12.2%	19.8%			
With family members	68.8%	72.6%				66.7%	73.0%			
With friends, in hostels, or in unstable homes	4.7%	2.7%				5.5%	2.7%			

**Table 2:** Differences between pathological gamblers with and without lifetime and current psychiatric disorders in gambling and clinical characteristics.

Variable	Lifetime psychiatric disorders					Current psychiatric disorders				
	With MI (*N* = 128) mean (SD)	Without MI (*N* = 73) mean (SD)	*χ* ^2^	df	*P*	With MI (*N* = 90) mean (SD)	Without MI (*N* = 111) mean (SD)	*χ* ^2^	df	*P*
Gambling characteristics										
Age of first gambling	20.0 (8.1)	19.6 (6.2)	−0.378	199	0.706	20.3 (8.5)	19.5 (6.6)	−0.773	199	0.441
Presence of debt (%)	82.0%	76.7%	0.825	1	0.364	86.7%	74.8%	4.409	1	0.036
ASI-G^a^ composite score	0.42 (0.21)	0.32 (0.17)	−3.174	199	0.002	0.45 (0.22)	0.33 (0.17)	−4.351	199	0.000

Clinical characteristics										
Brief symptom inventory										
Somatization	0.78 (0.78)	0.38 (0.47)	−3.973	199	0.000	0.87 (0.83)	0.44 (0.53)	−4.429	199	0.000
Obsessive-compulsive	1.47 (0.82)	0.90 (0.78)	−4.765	199	0.000	1.60 (0.83)	0.99 (0.77)	−5.371	199	0.000
Interpersonal sensitivity	1.39 (0.84)	0.78 (0.59)	−5.453	199	0.000	1.53 (0.85)	0.88 (0.65)	−6.143	199	0.000
Depression	1.61 (1.02)	0.85 (0.79)	−5.540	199	0.000	1.79 (0.99)	0.96 (0.87)	−6.363	199	0.000
Anxiety	1.25 (0.95)	0.72 (0.71)	−4.154	199	0.000	1.44 (0.98)	0.75 (0.69)	−5.766	199	0.000
Hostility	0.94 (0.76)	0.61 (0.69)	−3.061	199	0.003	1.02 (0.78)	0.67 (0.69)	−3.343	199	0.001
Phobic anxiety	0.74 (0.74)	0.31 (0.47)	−4.439	199	0.000	0.82 (0.80)	0.39 (0.51)	−4.660	199	0.000
Paranoid ideation	0.87 (0.74)	0.48 (0.55)	−3.865	199	0.000	0.90 (0.77)	0.58 (0.60)	−3.331	199	0.001
Psychoticism	1.14 (0.84)	0.62 (0.61)	−4.719	199	0.000	1.25 (0.86)	0.71 (0.66)	−5.044	199	0.000
GSI^a^	1.15 (0.71)	0.64 (0.53)	−5.398	199	0.000	1.27 (0.72)	0.71 (0.56)	−6.135	199	0.000
PST^ a ^	32.4 (13.6)	21.8 (14.7)	−5.153	199	0.000	34.0 (13.6)	24.0 (15.4)	−5.032	199	0.000
PSDI^ a ^	1.78 (0.61)	1.44 (0.39)	−4.194	199	0.000	1.89 (0.64)	1.46 (0.41)	−5.612	199	0.000
Psychosocial impairment										
LIFE-RIFT^ a ^	11.93 (3.28)	8.96 (2.60)	−6.444	188	0.000	12.72 (3.11)	9.30 (2.80)	−8.004	188	0.000
Severity of nicotine problem										
FTND^ a ^	4.56 (3.00)	2.03 (2.46)	−3.979	93	0.000	5.00 (2.86)	2.39 (2.70)	−4.558	93	0.000

^a ^ASI-G: Addiction Severity Index—Gambling Section; GSI: General Severity Index; PST: Positive Symptom Total Score; PSDI: Positive Symptom Distress Index; LIFE-RIFT: Longitudinal Interval Follow-up Evaluation-Range of Impaired Functioning Tool; FTND: Fagerstrom Test for Nicotine Dependence.

**Table 3:** Prevalence rates of lifetime and current comorbid psychiatric disorders in 201 pathological gamblers.

Variable	Lifetime psychiatric disorders					Current psychiatric disorders				
	*N*	%	PG first^a^	MI first^b^	Same year^c^	*N*	%	PG first^a^	MI first^b^	Same year^c^
Any psychiatric disorder	128	63.7	34.9%	55.8%	9.3%	90	44.8	30.0%	58.9%	11.1%
Mood disorders										
Any mood disorder	59	29.4	62.7%	23.7%	13.6%	43	21.4	65.9%	20.5%	13.6%
Major depressive disorder	43	21.4	60.5%	25.6%	14.0%	30	14.9	63.3%	23.3%	13.3%
Bipolar disorder	4	2.0	75.0%	0%	25.0%	2	1.0	50.0%	0%	50.0%
Dysthymic disorder	13	6.5	76.9%	15.4%	7.7%	13	6.5	76.9%	15.4%	7.7%
Schizophrenia spectrum disorder										
Any schizophrenia spectrum disorder	5	2.5	40.0%	60.0%	0%	3	1.5	66.7%	33.3	0
Schizophrenia	4	2.0	50.0%	50.0%	0%	3	1.5	66.7%	33.3	0
Substance use disorder										
Any substance use disorder	62	30.8	15.9%	74.6%	9.5%	46	22.9	8.7%	78.3	13.0
Alcohol abuse or dependence	23	11.4	30.4%	60.9%	8.7%	16	8.0	25.0%	62.5	12.5
Nicotine dependence	49	24.4	16.3%	75.5%	8.2%	41	20.4	12.2%	78.0	9.8
Any drug abuse or dependence	10	5.0	50.0%	40.0%	10.0%	9	4.5	55.6%	33.3	11.1
Anxiety disorder										
Any anxiety disorder	19	9.5	36.8%	57.9%	5.3%	17	8.5	35.3%	58.8	5.9
Panic disorder	5	2.5	50.0%	25.0%	25.0%	4	2.0	66.7%	25.0	33.3
Agoraphobia	2	1.0	100.0%	0%	0%	1	0.5	100.0%	0	0
Social phobia	1	0.5	0%	100.0%	0%	0	0	0	0	0
Specific phobia	8	4.0	25.0%	75.0%	0%	1	0.5	16.7	86.3	0
Obsessive compulsive disorder	4	2.0	0%	100.0%	0%	1	0.5	0	100.0	0
Posttraumatic stress disorder	4	2.0	50.0%	50.0%	0%	4	2.0	50.0	50.0	0
Generalized anxiety disorder	4	2.0	100.0%	0%	0%	4	2.0	100.0	0	0
Adjustment disorder	42	20.9	64.3%	21.4%	14.3%	28	13.9	60.7	21.4	17.9

^a^“PG First” refers to onset of pathological gambling preceded onset of comorbid psychiatric disorder.

^b^“MI First” refers to onset of comorbid psychiatric disorder preceded onset of pathological gambling.

^c^“Same Year” refers to both pathological gambling and comorbid psychiatric disorder developed in the same year.

**Table 4:** Number of lifetime and current comorbid psychiatric disorders for pathological gamblers seeking treatment.

Variables	Lifetime psychiatric disorders (*N* = 128)		Current psychiatric disorders (*N* = 90)	
Number of psychiatric disorders	*N *	%	*N *	%
1	73	57.0	69	76.7
2	33	25.8	12	13.3
3 or above	22	17.2	9	10.0

	Mean = 1.70; SD = 1.05		Mean = 1.36; SD = 0.72	

**Table 5:** Lifetime and current psychiatric disorder and demographic predictors of gambling severity.

Variables	Lifetime psychiatric disorders (*N* = 128)		Current psychiatric disorders (*N* = 90)	
	Beta	*P*	Beta	*P*
Presence of lifetime psychiatric disorder	0.239**	0.001	0.300***	0.000
Age (20 or below as reference)				
21–30	0.094	0.741	0.138	0.622
31–40	0.271	0.412	0.304	0.350
41–50	0.282	0.445	0.344	0.342
50 or above	−0.174	0.052	−0.188	0.033
Gender (male as reference)				
Female	0.129	0.102	0.100	0.196
Marital status (single as reference)				
Married or cohabitated	−0.050	0.571	−0.041	0.642
Divorced or separated or widowed	−0.014	0.874	−0.034	0.693
Education (lower secondary school or below as reference)				
Upper secondary school	−0.197*	0.040	−0.183	0.052
Sixth form or above	−0.106	0.288	−0.090	0.361
Economic status (employed as reference)				
Retired/homemaker/student	0.036	0.722	−0.032	0.708
Unemployed	0.206	0.732	0.016	0.876
Income (5,000 or below as reference)				
5,001–10,000	0.206	0.087	0.190	0.106
10,001–15,000	0.170	0.180	0.159	0.199
15, 001 or above	0.239	0.085	0.192	0.156

	Adjusted *R* ^2^ = 0.073; *F*(15,185) = 2.048*		Adjusted *R* ^2^ = 0.104; *F*(15,185) = 2.543**	

Note. *P < 0.05, **P < 0.01, ***P < 0.001. Beta is the standardized regression coefficient.
